# Genetics of Coronary Artery Disease in Taiwan: A Cardiometabochip Study by the Taichi Consortium

**DOI:** 10.1371/journal.pone.0138014

**Published:** 2016-03-16

**Authors:** Themistocles L. Assimes, I. -T. Lee, Jyh-Ming Juang, Xiuqing Guo, Tzung-Dau Wang, Eric T. Kim, Wen-Jane Lee, Devin Absher, Yen-Feng Chiu, Chih-Cheng Hsu, Lee-Ming Chuang, Thomas Quertermous, Chao A. Hsiung, Jerome I. Rotter, Wayne H.-H. Sheu, Yii-Der Ida Chen, Kent D. Taylor

**Affiliations:** 1 Department of Medicine, Stanford University School of Medicine, Stanford, California, United States of America; 2 Division of Endocrine and Metabolism, Department of Internal Medicine, Taichung Veterans General Hospital, Taichung, Taiwan; 3 Cardiovascular Center and Division of Cardiology, Department of Internal Medicine, National Taiwan University Hospital, National University College of Medicine, Taipei, Taiwan; 4 Institute for Translational Genomics and Population Sciences, Los Angeles Biomedical Research Institute at Harbor-UCLA Medical Center, Torrance, and Department of Pediatrics, University of California Los Angeles, Los Angeles, California, United States of America; 5 Department of Medical Research, Taichung Veterans General Hospital, Taichung, Taiwan; 6 HudsonAlpha Institute for Biotechnology, Huntsville, Alabama; 7 Institute of Population Health Sciences, Division of Biostatistics and Bioinformatics, National Health Research Institutes, Zhunan Town, Miaoli County, Taiwan; 8 Division of Endocrine and Metabolism, Department of Internal Medicine, National Taiwan University Hospital, Taipei, Taiwan; Hospital Authority, CHINA

## Abstract

By means of a combination of genome-wide and follow-up studies, recent large-scale association studies of populations of European descent have now identified over 46 loci associated with coronary artery disease (CAD). As part of the TAICHI Consortium, we have collected and genotyped 8556 subjects from Taiwan, comprising 5423 controls and 3133 cases with coronary artery disease, for 9087 CAD SNPs using the CardioMetaboChip. We applied penalized logistic regression to ascertain the top SNPs that contribute together to CAD susceptibility in Taiwan. We observed that the 9p21 locus contributes to CAD at the level of genome-wide significance (rs1537372, with the presence of C, the major allele, the effect estimate is -0.216, standard error 0.033, p value 5.8x10^-10^). In contrast to a previous report, we propose that the 9p21 locus is a single genetic contribution to CAD in Taiwan because: 1) the penalized logistic regression and the follow-up conditional analysis suggested that rs1537372 accounts for all of the CAD association in 9p21, and 2) the high linkage disequilibrium observed for all associated SNPs in 9p21. We also observed evidence for the following loci at a false discovery rate >5%: SH2B3, ADAMTS7, PHACTR1, GGCX, HTRA1, COL4A1, and LARP6-LRRC49. We also took advantage of the fact that penalized methods are an efficient approach to search for gene-by-gene interactions, and observed that two-way interactions between the PHACTR1 and ADAMTS7 loci and between the SH2B3 and COL4A1 loci contribute to CAD risk. Both the similarities and differences between the significance of these loci when compared with significance of loci in studies of populations of European descent underscore the fact that further genetic association of studies in additional populations will provide clues to identify the genetic architecture of CAD across all populations worldwide.

## Introduction

Recent large-scale association studies of populations of European descent have now identified over 46 loci associated with coronary artery disease (CAD)[1–3]. In order to achieve this result, the CARDIoGRAM consortium performed meta-analyses on numerous genome-wide association datasets with over 22,000 CAD cases and over 64,000 controls, with subsequent meta-analyses of follow-up studies with over 63,000 CAD cases and 130,000 controls employing the Illumina CardioMetaboChip. The CARDIoGRAM Consortium contributed the top loci from their genome-wide analyses to the design of this chip and, along with other consortia studying cardio-metabolic traits, were able to achieve these large numbers because of the “economy of scale” made possible by the price reduction available due to the large number of chips manufactured for this combined effort [4]. Since the initial genome-wide association studies of CAD in 2007 [5, 6], the greatest contribution to CAD remains the 9p21 locus. The features of this locus include the CDKN2A and CDKN2B genes, a CDKN2B-antisense RNA known as ANRIL, microRNA 384, and numerous binding sites for transcription factors as identified by ENCODE [7]. While a recent sequencing study of the 9p21 region in the Framingham Cohort demonstrated associations with higher risk for myocardial infarction, higher coronary calcium scores, and larger aortic diameters, no obviously functional polymorphisms were found; however, variants were associated with gene expression of a short form of ANRIL and for the gene CDK2NB [8].

With respect to populations of Chinese descent, a meta-analysis of two genome-wide association studies of Han subjects from Beijing by the CARDIoGRAM consortium (over 1500 cases and over 5000 controls), followed by a replication study (over 15,000 cases and over 11,000 controls), replicated four of the European loci, 9p21, PHACTR1, TCF21, and C12orf51 (now known as HECTD4), and identified four additional four loci, TTC32-WDR35, GUCY1A3, the MHC, and ATP2B1 [9]. This study proposed that there were two association signals in the 9p21 locus because: 1) a conditional analysis demonstrating additional association with several SNPs after controlling for the main effect, and 2) differences in the linkage disequilibrium pattern of these associated SNPs were observed between their samples and samples of European descent from the International HapMap Project.[10]

As amply demonstrated by these and other studies of non-Mendelian or complex genetic traits, the genome-wide association study represents one of the most powerful tools for the identification of the genetic contribution of individual SNPs to specific phenotypes. Most of these studies have been limited to analyses of the association of phenotype with individual genetic loci or single-nucleotide polymorphisms (SNPs), taken one at a time. This is because applying multivariate regression to such a large dataset, with the goals of investigating the contribution of all SNPs to phenotype and of constructing models of the genetic architecture of the phenotype, becomes computationally impossible with the large number of SNPs to be considered; the requisite matrix inversion procedures or linear equations scale with the number of SNPs to the power of three [11]. One means to circumvent these problems is to employ the use of a regression penalty that shrinks most of the coefficients (“betas”) to zero during the selection of the model. Such a penalty may be estimated by cross-validation methods. Furthermore, penalized methods make it possible to explore SNP by SNP interactions and so may provide clues to the genetic architecture of the phenotype [12].

For the study of the genetics of CAD in Taiwan, we took advantage of the fact that the CAD SNPs identified in populations of European descent were incorporated into the design of the Illumina CardioMetaboChip [4]. This design provided a cost-effective means to genotype known CAD SNPs. Through our academic collaboration, we have collected phenotypic data and DNA from a large cohort of subjects with coronary artery disease and controls without disease across the island of Taiwan. We genotyped this cohort with the CardioMetaboChip and applied the method of penalized logistic regression; using this approach, we were able to identify gene-by-gene interactions contributing to CAD in this population.

## Methods

### Subjects

The TAICHI Consortium is a collaborative study in Taiwan, with the aims to genotype a large number of Taiwanese Chinese subjects with the Illumina CardioMetaboChip and to identify genetic determinants of atherosclerosis- and metabolic-related traits [13–20]. Academic centers participating include Taichung Veteran’s General Hospital, Tri-Service General Hospital and the National Taiwan University Hospital, and the National Health Research Institute in Taiwan for subject ascertainment and phenotyping. A total of 8556 subjects, 3133 cases with coronary artery disease, 5423 controls without disease, were recruited in this report (defined below; see also Table 1).

**Table 1:** Characteristics of Subjects.

	No CAD		CAD	
	Male	Female	Male	Female
Number of subjects	2728	2695	2325	808
Age (mean, yr)	66	65	65	67
BMI (mean, kg/m^2^)	24.7	24.8	25.6	25.5
SBP (mean, mmHg)	128	130	130	134
DBP (mean, mmHg)	74	72	75	74
Total cholesterol (mean, mg/dl)	185	197	179	190
LDL-cholesterol (mean, mg/dl)	114	118	107	111
HDL-cholesterol (mean, mg/dl)	45	52	42	45
Triglycerides (mean, mg/dl)	143	141	149	172

Age, BMI, blood pressure, and serum lipids are significantly different between case/control status and between gender at p<0.001 by analysis of variance.

Institutional Review Boards at all participating centers have approved this study: Taichung Veteran’s General Hospital, Tri-Service General Hospital, the National Taiwan University Hospital, and the National Health Research Institute of Taiwan. Written informed consent was obtained from each participant and is on file in Taiwan. All data and samples that are transferred to the Los Angeles Biomedical Research Institute in the US are identified by codes so that all personal identifying information is kept in Taiwan. The Institutional Review Board (IRB) of the Los Angeles Biomedical Research Institute has approved collection and handling of data and samples from Taiwan as well as molecular studies of the samples including genotyping, all data analyses, and publication of results. The IRB’s of the Hudson-Alpha Research Institute and Stanford University have given approval to this project as exempt from the regulations at 45 CFR 46 or 21 CFR56.

### Phenotype

#### CAD

Patients admitted to the several Hospital Cardiovascular Departments were enrolled. Subjects were defined as CAD positive with one of the following criteria: 1) history of myocardial infarction; 2) history of coronary artery bypass graft (CABG) or percutaneous coronary angioplasty (PTCA) and or stenting; or 3) at least 1 major vessel with stenosis ≥ 50% demonstrated by angiography. Age was defined as age of event, graft or angioplasty, or angiography.

#### Controls

Subjects were enrolled from an epidemiology study and from the several Hospital Endocrinology and Metabolism Departments either as outpatients or as their family members. Subjects with history of CAD were excluded. Age was defined as age of recruitment.

### Genotypes

#### Chip

Genotyping was performed using the Illumina CardioMetaboChip v1.0 (Illumina, San Diego, CA) following the manufacturer’s protocol [21, 22] in a total of 11,582 subjects using DNA purified by column from whole blood (Qiagen, Valencia, CA). The chip has been described elsewhere [4]. Calling of genotypes was performed using the Illumina Genome Studio software and both samples and SNPs were removed with call rates less than 98%.

#### Quality Control

Because the set of subjects includes family members, subjects were removed for relatedness and cryptic relatedness with PI-Hat values >0.125. Population outliers were defined as those subjects with eigenvalues from principal components 1, 2, and 3 greater than 3 standard deviations from the mean and were removed after calculating principal components using standard methods [23]. Only subjects with age between 40 and 90 years were then retained. Additional SNPs were removed if the minor allele frequency < 5% or if the p-value from the Hardy-Weinberg test of allele frequencies in controls <0.05. The results from 8556 subjects are therefore included in this report.

#### CAD SNPs

“CAD SNPs” were selected from the 23 fine-mapping regions and GWAS hits contributed by the Cardiogram Consortium to the original design of the CardioMetaboChip[4]. This list and other CardioMetaboChip annotation files are available from the Abecasis group at http://csg.sph.umich/kang/MetaboChip. The results from 9087 SNPs are reported here.

### Statistical Analyses

#### Penalized logistic regression

Penalized logistic regression was employed using the software MENDEL v14 [24] available from http://www.genetics.ucla.edu/software/mendel with age, gender, and eigenvalues from principal components 1 and 2 added as covariates. As applied to this study, lasso penalized regression is a method for continuous model selection on all of the CAD SNPs and covariates, together and analyzed at the same time, and is of particular value when the number of SNPs exceeds the number of subjects, as in the standard GWAS design.[11] The order that each SNP is added to the statistical model is correlated with its individual significance, and the lasso method eliminates SNPs that enter with low explanatory power. The result of a lasso penalized regression of this dataset may be considered to have studied the association of all CAD SNPs on the CardioMetaboChip.

Two-way interactions were also explored by penalized lasso logistic regression within the top 100 associated SNPs in order to reduce the number of interactions to be computed using the Mendel software.

#### Other tests

As implemented in R software, the Fisher exact test, calculation of Odds Ratios, logistic regression, and the Cochran-Mantel-Haenszel test were used to explore the effects of gender in the association of CAD, both within and across gender, and across genotypes [25, 26]. Conditional analysis was performed using PLINK v1.9 [27, 28] with covariates as listed in the legends to the tables. “Locus Zoom” plots were drawn using the website at the University of Michigan [29]. Linkage disequilibrium was assessed using Haploview v4.[30] Control data from the Thousand Genomes Project were used as indicated.[31] Power for detecting an association was computed using QUANTO (available from http://biostats.usc.edu/Quanto.html).

## Results

### Dataset

Herein we report observations from 8556 Taiwan subjects (3133 cases and 5423 controls) and 9087 coronary artery disease (CAD) SNPs genotyped using the Illumina CardioMetaboChip.

Characteristics of the subjects by gender and by case/control status are given in Table 1. As expected, cases and controls were significantly different for body mass index (BMI), serum lipids, and blood pressure (p anova <0.001).

### Association Study

#### Penalized logistic regression

The 9087 coronary artery disease (CAD) SNPs identified by the CARDIoGRAM Consortium for populations of European descent were tested for association with CAD in Taiwan using a penalized logistic regression method. This method allows consideration of the contribution of all SNPs together and is appropriate for a dataset with more SNPs than subjects. With age, gender, and the eigenvalues from principal components 1 and 2 as covariates, the p-values and effect estimates are given in Table 2. Loci associated with CAD with a significance less than a false discovery rate of 5% were: 9p21, SH2B3, ADAMTS7, PHACTR1, GGCX, HTRA1, COL4A1, and LARP6-LRRC49. Furthermore, the 9p21 locus was significantly associated with CAD by the “genome-wide” criteria of 5x10^-8^ (rs1537372, with the presence of C, the major allele, the effect estimate is -0.216, standard error 0.033, p value 5.8x10^-10^).

**Table 2:** Association of SNPs with Coronary Artery Disease by Penalized Logistic Regression.

SNP ID	Locus	Chr	Position	P Value	Effect	Standard Error	Major Allele	Allele Freq. Cases	Allele Freq. Controls
rs1537372	9p21	9	22,093,183	5.7x10^-10^	-0.216	0.033	C	0.50	0.54
rs79105258	SH2B3	12	110,202,614	2.5x10^-5^	-0.140	0.036	C	0.70	0.72
rs79265682	ADAMTS7	15	76,829,811	3.8x10^-5^	0.138	0.034	G	0.62	0.58
rs9349379	PHACTR1	6	13,011,943	4.4x10^-5^	0.153	0.037	G	0.73	0.70
rs6738645	GGCX	2	85,636,639	6.3x10^-5^	-0.130	0.034	A	0.60	0.63
rs2268344	HTRA1	10	124,234,959	0.00012	-0.129	0.034	A	0.58	0.61
rs2289800	COL4A1	13	109,622,824	0.00019	-0.132	0.036	G	0.68	0.71
rs11072221	LARP6	15	68,919,620	0.00023	-0.121	0.033	C	0.50	0.53

Effect estimates in null model: Grand Mean, -0.68; Age, 0.049; Gender, -0.52. Eigenvalues from Principal Components 1, 2, and 3 were included as covariates. False discovery rate < 5% corresponds to a p-value of 0.0004. Positions of SNPs are GRCh37/hg19.

#### Conditional analysis

**Fig 1:**
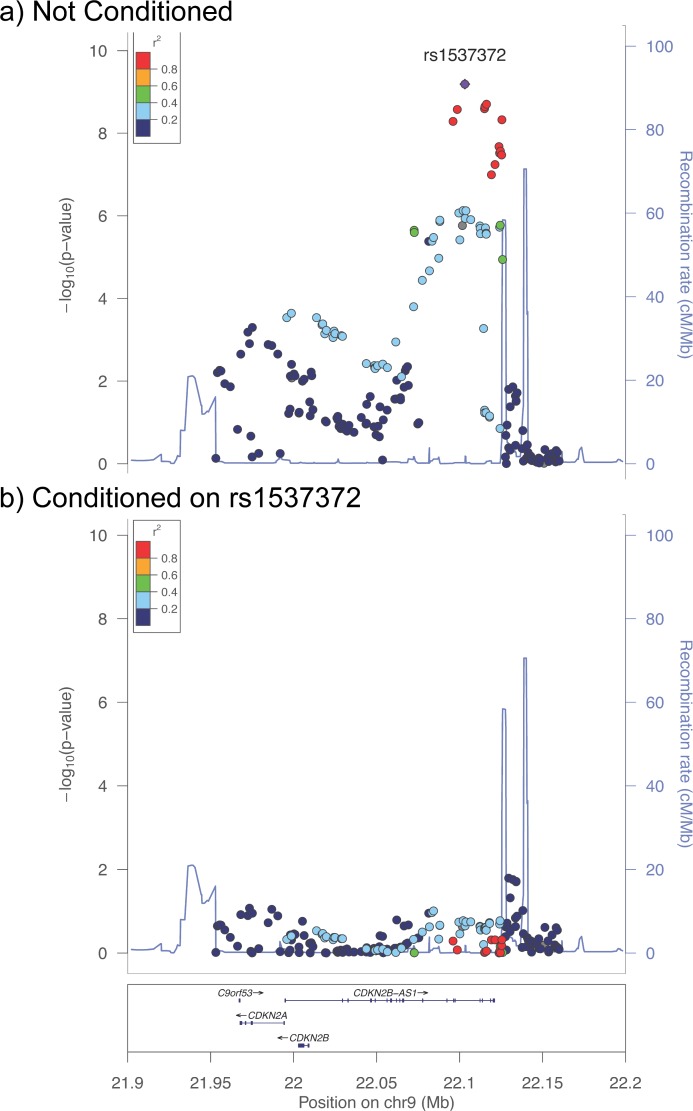
Conditional Analysis of the 9p21 Region. “Locus Zoom” plots [29] of the 9p21 region demonstrating conditional analysis of the top SNP rs1537372 using logistic regression adjusted for age, gender, and the eigenvalues from principal components 1 and 2. Linkage disequilibrium of SNPs with rs1537372 is shown by the color of the points and is based on data from the Asian subjects in the Thousand Genomes Project. a) Association of SNP with coronary disease; b) Association of SNP with coronary disease conditioned on rs1537372 (rs1537372 added as an additional covariate).

As a check of the result of the penalized logistic regression, we investigated the fact that only a single SNP in the 9p21 region was retained. A “conventional,” or single-SNP logistic regression of the 9p21 region was conducted with age, gender, and the eigenvalues from principal components 1 and 2 as covariates; the results without and with conditioning on the 9p21 SNP that was retained by the penalized logistic regression, rs1537372, are compared in Fig 1 (“Locus Zoom” plot; without rs1537372 as a covariate, Fig 1A; with rs1537372 as an additional covariate, Fig 1B). No additional association was detectable in the 9p21 region when conditioning on rs1537372, thus providing additional support for the retention of one 9p21 SNP in the penalized logistic regression. Furthermore, SNPs with significant associations in this conventional logistic regression are in high linkage disequilibrium with each other as determined in data from Asian subjects in the Thousand Genomes Project (Fig 1, color of data points; detail in Fig).

In order to place our work within the context of the previous study of CAD in a Chinese population,[9] we show a detail of this conditional analysis for the previous reported SNPs rs10757274, rs1333049, rs9632884, and rs1333042 (S1 Table). The association of each of these four SNPs is abolished by conditioning on the top SNP in this report, rs1537372. We also calculated the power for an association with 3130 cases and 5423 controls at a significance level of 0.05 using the program QUANTO. At the observed minor allele frequency (MAF) of rs1333042 (0.32) and of rs10757274 (0.48), we have 80% or 85% power, respectively, to detect an association with a relative risk of 1.10, and at the MAF of rs9632884 (0.27), we have 76% power to detect an association with a relative risk of 1.10. Thus, we had sufficient power to detect additional associations in 9p21 if such had existed in our dataset.

### Interaction Study

Since penalized methods are also efficient for exploring two-way interactions, we explored possible interactions among the 100 SNPs with the highest association with CAD (Table 3). Please note that this is an analysis separate and secondary to the analysis presented in Table 2. Differences in p values between the two tables result from the absence or presence of two-way interactions. The result suggested the presence of SNP by SNP interactions between PHACTR1 and ADAMTS7, and between COL4A1 and SH2B3.

**Table 3:** Possible SNP by SNP Interactions Contributing to Coronary Artery Disease in Taiwan by Penalized Logistic Regression.

Individual SNP or SNP x SNP Interaction	P Value	Effect Estimate	SNPs	
9p21	8.5x10^-10^	-0.208	rs1537372	
PHACTR1 x ADAMTS7	1.9x10^-7^	-0.104	rs9349379	rs79265862
COL4A1 x SH2B3	2.5x10^-7^	-0.137	rs2289800	rs79105258
PHACTR1	2.8x10^-5^	0.131	rs9349379	
ADAMTS7	3.0x10^-5^	0.055	rs79265862	
GGCX1	5.4x10^-5^	-0.128	rs6738645	
HTRA1	0.00015	-0.122	rs2268344	

Effect estimates in null model and covariates included in the analysis, are the same as in legend to Table 1.

These interactions were studied further using frequency tables (Tables 4 and 5). For the interaction between PHACTR1 and ADAMTS7, the Odds Ratio for CAD was highest for subjects with both the PHACTR1 GG genotype and the ADAMTS7 GG or GA genotype (Table 4). A Cochran-Mantel-Haenszel test across all genotype combinations confirmed the interaction between these two loci that was observed in the penalized logistic regression analysis (CMH p value = 0.00015). The highest Odds Ratio for CAD was therefore observed in those subjects homozygous for the major allele at both loci. For the interaction between SH2B3 and COL4A1, the Odds Ratio for CAD was highest for subjects with both the SH2B3 AA genotype and the COL4A1 GG genotype (Table 5; Cochran-Mantel-Haenszel test p value = 0.00047). The highest Odds Ratio for CAD was therefore seen in those subjects homozygous for the minor allele for the SH2B3 locus and the major allele for the COL4A1 locus.

**Table 4:** Interaction of PHACTR1 and ADAMTS7.

	ADAMTS7 rs79265862 Genotype														
PHACTR1 rs9349379 Genotype	GG					GA					AA				
	Control		Case		OR	Control		Case		OR	Control		Case		OR
	N*	freq	N	freq		N	freq.	N	freq		N	freq	N	freq	
GG	898	0.487	654	0.550	1.67	1285	0.493	753	0.509	1.42	467	0.481	228	0.494	0.952
GA	759	0.411	454	0.382	1.22	1070	0.410	623	0.421	1.00	425	0.438	195	0.421	1.064
AA	188	0.102	82	0.069	**	253	0.097	104	0.070	**	78	0.080	40	0.086	**
Fisher Test for 3x3	2.7 x 10^−4^					0.014					ns				

* N, number; freq, frequency

** assigned OR = 1

ns: Not significant at p<0.05.

Cochran-Mantel-Haenszel test across the three genotypes, p = 0.00015.

**Table 5:** Interaction of SH2B3 and COL4A1.

	COL4A1 rs2289800 Genotype														
SH2B3 rs79105258 Genotype	GG					GA					AA				
	Control		Case		OR	Control		Case		OR	Case		Control		OR
	N*	Freq	N	Freq		N	Freq	N	Freq		N	Freq	N	freq	
CC	1379	0.508	660	0.454	**	1109	0.497	631	0.469	**	208	0.438	149	0.449	**
CA	1128	0.415	631	0.434	1.17	912	0.409	573	0.426	1.10	215	0.453	150	0.458	0.99
AA	210	0.077	164	0.113	1.61	210	0.094	142	0.105	1.18	52	0.109	33	0.099	0.88
Fisher Test for 3x3	6.9x10^-5^					ns					ns				

* N, number; freq, frequency

** assigned OR = 1

ns: Not significant at p<0.05.

Cochran-Mantel-Haenszel test across the three genotypes, p = 0.00047.

## Discussion

In this study we applied penalized logistic regression to a dataset composed of Taiwanese CAD subjects and controls genotyped for CAD SNPs identified by the CARDIoGRAM Consortium and included in the design of the Illumina CardioMetaboChip. We observed that the major genetic contribution to CAD in the Taiwanese is represented by a single association at the 9p21 locus. This association was of “genome-wide” significance at <5x10^-8^ in our dataset (Table 2). Thus, the strongest genetic signal for CAD in Taiwanese is very similar or identical to the strongest GWAS signal observed in populations of European descent [1] and to the signal identified by a candidate gene approach [15]. The conclusion that this association represents a single genetic contribution was suggested by our observations that: 1) the penalized logistic regression retained only one SNP from this locus (Table 2), 2) SNPs with significant associations in a conventional logistic regression analysis were in high linkage disequilibrium with each other (Fig 1A and S1 Fig), and 3) conditioning on this single SNP reduced all other associations in this region to non-significance (Fig 1B), and 4) we had sufficient power to detect additional association if such had existed in our dataset.

Our observations supporting a single genetic contribution to CAD susceptibility in the 9p21 region contrast with the previous report of a CAD GWAS in a Chinese population [9]. That study ascertained subjects from Beijing and from northern provinces in China and reported: 1) a difference in linkage disequilibrium (LD) structure between their sample and populations of European descent, and 2) a conditional analysis supporting two independent genetic signals from SNPs rs1333042 and rs10757274. Our dataset contains, in addition to our top SNP, rs1537372, high quality genotypes for rs1333042 and rs10757274, plus two others mentioned in the previous report, rs1333049 and rs9632884. We observe all five SNPs to be in high linkage disequilibrium with each other with an r^2^ value > 0.87 in data from Asian subjects in the Thousand Genomes Project (Panel A in S1 Fig), in data from European subjects in the Thousand Genomes Project (Panel B in S1 Fig) as well as in our own data from Taiwan (Panel C in S1 Fig). We therefore do not observe the differences in linkage disequilibrium structure reported previously. Furthermore, we observed no additional association upon conditioning on our top SNP, rs1537372 (S1 Table). We therefore think that the contrast between our results and those of Lu and co-workers are due to differences in the populations studied: the previous work combined subjects from Beijing and the northern provinces of China, while our work studied subjects from the island of Taiwan.

We identified additional suggestive loci, significant at a false discovery rate less than 0.05; in order of importance these were: SH2B3, ADAMTS7, PHACTR1, GGCX, HTRA1, COL4A1, LARP6-LRRC49.

By allowing two-way interactions in the penalized logistic regression, we observed an interaction between the major alleles of the PHACTR1 and ADAMTS7 loci contributes to CAD in Taiwan (Table 4). In addition to association with CAD, PHACTR1 (phosphatase and actin regulator 1; dbGENE 221692) has been associated with cervical artery dissection association [32] and shown to play a role in regulation of endothelial cells [33]. ADAMTS7 (a disintegrin and metalloproteinase with thrombospondin type 1 motif; dbGENE 11173) has been recently shown to affect the migration of smooth muscle cells [34, 35]. While PHACTR1 is also expressed in smooth muscle cells [35], further work will be required to unravel the relationship between these two genes in CAD risk.

We also observed that the SH2B3 and COL4A1 contribute to CAD through an interaction, suggesting that the minor allele of SH2B3 contributes to CAD only in the presence of the major allele of the COL4A1 locus (Table 5). SH2B3 (SH2B adaptor protein 3; dbGENE 10019) is involved in cytokine signaling pathways and COL4A1 (collagen type 4 alpha 1; dbGENE 1282) has also been implicated in cerebral small vessel disease [36]. Both genes are up-regulated over 5-fold as endothelial cells change from normal to the atherosclerotic disease state in a mouse model of atherosclerosis [35].

While the mathematical advantages of penalized logistic regression methods have been discussed elsewhere,[11, 12, 37] we have compared the results of the penalized logistic regression reported here with a standard logistic regression in order to draw practical conclusions on the use of this method (S2 Table; age, gender, and the eigenvalues from principal components 1 and 2 as covariates; top 100 results are listed; Plink v1.90). First, the p-values for a given SNP are of the same magnitude and very close in value between the two analyses. Thus, p-values reported using penalized logistic regression will be comparable to those obtained with standard logistic regression. Second, the penalized logistic regression result readily suggested that all of the 50 9p21 associations represented a single association signal, since no other 9p21 loci were identified by this method. Third, the interaction analysis identified two SNP by SNP interactions within these top 100 SNPs.

Our conclusions on the genetic architecture of coronary artery disease in Taiwan are limited by several factors. First, our sample size of 3133 cases and 5423 controls, while among the largest reported for a population of Chinese descent, yet remains modest when compared with the sample sizes reported for populations of European descent by the CARDIoGRAM consortium. Aside from the genome-wide significance of 5x10^-8^ observed for the 9p21 locus, the results with a significance of a false discovery rate less than 5% should be considered tentative for Taiwan. However, it should be noted in this context that all loci tested in this report were already established CAD loci and this fact further supports the conclusions presented here. The next step to be taken by us will be to collaborate with other groups studying CAD genetics of East Asians with the aim of confirming the conclusions presented here. Second, since our SNPs were selected based on GWAS results from European populations as incorporated in the CardioMetaboChip and not based on coverage of the entire genome, it is likely that there are genetic effects contributing to the genetic architecture of CAD in Taiwan not yet detected. The next step to be taken by us will be to genotype a SNP set selected for genome-wide coverage of East Asian populations, in addition to collaborating with other investigators. Third, while we did identify 2 SNP by SNP interactions, the interaction analysis was limited to the study of two-way interactions. We expect to explore higher order complexities contributing to CAD in Taiwan in future studies with larger samples and more genotypes.

While all SNPs tested in this report were selected from meta-analyses by the CARDIoGRAM consortium for the design of the CardioMetaboChip, two of these loci, HTRA1 and LARP6-LRRC49, were not listed in the final tables of GWAS studies of CAD associations in Europeans [1] nor in Chinese from Beijing [9]. Furthermore, we did not observe an association with the SORT1 or ZC3HC1 loci, though both have been reported with effects equal or greater in significance to the PHACTR1 and ADAMTS7 loci in Europeans. While this observation could be the effect of our smalller sample size or differences in subject ascertainment, there may also be differences in the magnitude of effect size such that different loci are most important for CAD in different populations. These observations, however, underscore the value of continuing genome-wide association studies in populations of other than European descent; comparisons between populations as well as within different populations will provide findings relevant to the genetics of CAD in all populations.

## Supporting Information

S1 Fig Linkage Disequilibrium between Major SNPs in the 9p21 Region. Linkage disequilibrium between the top SNP in this report and the four coronary artery disease SNPs reported in Lu et al.[9] Figures prepared using Haploview v4.[30] Panel A) Data for subjects of Chinese descent from the Thousand Genomes Project;[31] Panel B) Data for subjects of European descent from the Thousand Genomes Project; Panel C) Data for Taiwan subjects in this report.
(TIFF)

S1 Table Conditional Analysis of Major SNPs in the 9p21 Region, Detail. Association assessed by logistic regression with age, gender, and the eigenvalues from Principal Components 1 and 2 as covariates; this table provides data from Fig 1 for SNPs reported by Lu et al.[9].
(XLSX)

S2 Table Comparison of Standard Logistic Regression with Penalized Logistic Regression. (XLSX)

S3 Table Comparison of standard logistic regression with penalized logistic regression. (XLSX)
